# Assessment of Effectiveness and Safety of Osimertinib for Patients With Intracranial Metastatic Disease: A Systematic Review and Meta-analysis

**DOI:** 10.1001/jamanetworkopen.2020.1617

**Published:** 2020-03-25

**Authors:** Anders W. Erickson, Priscilla K. Brastianos, Sunit Das

**Affiliations:** 1 Currently a student at Institute of Medical Science, Faculty of Medicine, University of Toronto, Toronto, Ontario, Canada; 2 Division of Hematology/Oncology, Department of Medical Oncology, Dana-Farber/Harvard Cancer Center, Harvard Medical School, Boston, Massachusetts; 3 Division of Neurosurgery, Department of Surgery, University of Toronto, St Michael’s Hospital, Toronto, Ontario, Canada; 4 Institute of Medical Science, Faculty of Medicine, University of Toronto, Toronto, Ontario, Canada

## Abstract

**Importance:**

Intracranial metastatic disease (IMD) is a serious and life-altering complication for many patients with cancer. Targeted therapy may address the limitations of current treatments as an additional agent to achieve intracranial disease control in some patients with IMD. Given the paucity of evidence regarding effectiveness, current guidelines have not made recommendations on the use of targeted therapy. Osimertinib mesylate is a mutant epidermal growth factor receptor (EGFR) inhibitor that can penetrate the blood-brain barrier and inhibit tumor cell survival and proliferation in patients with non–small cell lung cancer (NSCLC) with specific EGFR alterations.

**Objective:**

To assess the effectiveness and safety of osimertinib in the management of IMD.

**Data Sources:**

Studies were selected from MEDLINE and Embase databases from their inception to September 20, 2019, using the following search query: (*osimertinib* OR *mereletinib* OR *tagrisso* OR *tamarix* OR *azd9291*) AND (*brain metastases* OR *intracranial metastatic disease* OR *cns*).

**Study Selection:**

Studies reporting intracranial outcomes for patients with metastatic EGFR-variant NSCLC and IMD treated with osimertinib were included in this systematic review and meta-analysis. Among 271 records identified in the systematic review, 15 studies fulfilled eligibility criteria for inclusion in the meta-analysis.

**Data Extraction and Synthesis:**

Data were extracted from published studies and supplements. These data were pooled using a random-effects model. Risk of bias was assessed using the Cochrane risk of bias tool and the modified Newcastle-Ottawa Scale.

**Main Outcomes and Measures:**

Information extracted included study characteristics, intracranial effectiveness measures, and safety measures. Meta-analyses of proportions were conducted to pool estimates for central nervous system (CNS) objective response rate and CNS disease control rate.

**Results:**

Fifteen studies reporting on 324 patients were included in the meta-analysis. The CNS objective response rate was 64% (95% CI, 53%-76%; n = 195), and CNS disease control rate was 90% (95% CI, 85%-93%; n = 246). Included studies reported complete intracranial response rates of 7% to 23%, median best decrease in intracranial lesion size of −40% to −64%, and Common Terminology Criteria for Adverse Events (version 3.0) grade 3 or higher adverse event rates of 19% to 39%. Subgroup analyses did not reveal additional sources of heterogeneity.

**Conclusions and Relevance:**

Findings reported herein support a potential role for osimertinib in the treatment of patients with metastatic EGFR-variant NSCLC and IMD treated with osimertinib. Clinical decision makers would benefit from the inclusion of patients with IMD in future trials to identify factors that predict responses to targeted therapy.

## Key Points

**Question:**

What are the effectiveness and safety of osimertinib mesylate in the management of intracranial metastatic disease from non–small cell lung cancer with alterations in the epidermal growth factor receptor?

**Findings:**

Among 15 studies reporting on 324 patients in this systematic review and meta-analysis, central nervous system objective response rate and central nervous system disease control rate were calculated for comparison with reports for other targeted therapies in intracranial metastatic disease management. Common Terminology Criteria for Adverse Events (version 3.0) grade 3 or higher adverse event rates were consistent with or lower than other targeted therapies.

**Meaning:**

These findings support the use of osimertinib in intracranial metastatic disease management.

## Teaser

This systematic review and meta-analysis assesses the effectiveness and safety of osimertinib in the management of intracranial metastatic disease.

## Introduction

Brain metastases, or intracranial metastatic disease (IMD), are a serious and common complication of cancer, occurring in 20% of patients with primary disease.^1^ Patients with IMD have a 2-year survival rate of 8.1% and are likely to experience decreases in quality of life from neurologic symptoms associated with their disease or treatment.^2,3^ Interest exists in new therapeutic modalities for patients with IMD because of the limitations of available options, such as surgical resection and stereotactic radiosurgery, which are often reserved for patients with good performance status and low tumor burden, or whole-brain radiotherapy, which is associated with neurocognitive decline.^4,5^ Targeted therapies have been proposed to fill this gap, despite historical concerns that the benefit of systemic therapies as treatments for IMD has been limited by their inability to penetrate the blood-brain barrier (BBB). The National Comprehensive Cancer Network^6^ includes 2 targeted therapies in their most recent IMD treatment guidelines, but the Congress of Neurological Surgeons^7^ cites insufficient evidence to recommend the use of targeted therapies in IMD.

Osimertinib mesylate was recently approved in North America as a first-line tyrosine kinase inhibitor (TKI) therapy for treatment of patients with metastatic non–small cell lung cancer (NSCLC), the largest contributor to IMD, whose tumors have an epidermal growth factor receptor (EGFR) exon 19 deletion, exon 21 L858R substitution, or exon 20 T790M resistance substitution.^8,9^ Cancer cells with EGFR alterations have constitutive activity at that receptor, inducing cell survival and proliferation; EGFR-TKIs like osimertinib competitively inhibit an intracellular adenosine triphosphate–binding domain to prevent downstream signaling.^10^ Patients with NSCLC are recommended for genotype testing of their primary or secondary tumors to assess for the presence of EGFR alterations to evaluate their tumor sensitivity to EGFR-TKIs.^11^ However, 41% to 62% of NSCLC tumors with EGFR alterations develop T790M substitutions, conferring resistance to first-generation and second-generation EGFR-TKIs.^12^ Osimertinib is a third-generation EGFR-TKI that overcomes T790M alteration and is among several targeted therapies that have been considered for use in the prevention and management of IMD because of its ability to penetrate the BBB.^13,14^

Multiple trials have demonstrated the effectiveness of osimertinib in primary NSCLC, but only 2 randomized clinical trials (RCTs) to date (AURA3 [AZD9291 vs Platinum-Based Doublet-Chemotherapy in Locally Advanced or Metastatic Non–Small Cell Lung Cancer]^15^ and FLAURA [AZD9291 vs Gefitinib or Erlotinib in Patients With Locally Advanced or Metastatic Non–Small Cell Lung Cancer]^16^) have presented subgroup analyses comparing central nervous system (CNS) efficacy in osimertinib vs control groups. The CNS data from ongoing and recent phase 3 trials of osimertinib (ADAURA [AZD9291 vs Placebo in Patients With Stage IB-IIIA Non–Small Cell Lung Carcinoma, Following Complete Tumour Resection With or Without Adjuvant Chemotherapy],^17^ ASTRIS [Real World Treatment Study of AZD9291 for Advanced/Metastatic EGFR T790M Mutation NSCLC],^18^ and APOLLO [Open Label, Prospective Study to Investigate Efficacy and Safety of AZD9291 in BM [brain metastases] From NSCLC Patients With EGFR T790M]^19^) will provide additional information on effectiveness in patients with IMD. Recent review articles^12,20,21^ have addressed the role of osimertinib in IMD; however, no study to our knowledge has aggregated CNS effectiveness data across existing RCTs and single-arm studies. Although some current guidelines^6^ support the use of osimertinib in IMD management, other treatment guidelines^6,7^ specifically cite a lack of higher-level evidence, such as meta-analyses, in not making a recommendation on targeted therapies. To clarify the role of osimertinib in the management of IMD, an aggregate analysis of multiarm and single-arm studies of the CNS response to osimertinib was performed.

## Methods

### Search and Selection Criteria

In this systematic review and meta-analysis, a literature search was conducted on September 20, 2019, using the following search query in MEDLINE and Embase databases: (*osimertinib* OR *mereletinib* OR *tagrisso* OR *tamarix* OR *azd9291*) AND (*brain metastases* OR *intracranial metastatic disease* OR *cns*). Only articles and abstracts published in English were considered, and all years from database inception to the search date were included. Study authors were not contacted. Retrieved records were screened by abstract for reference to osimertinib as treatment for IMD. Case reports, case series, and review articles were excluded. Records reporting intracranial outcomes were included in the analysis. This study followed the Preferred Reporting Items for Systematic Reviews and Meta-analyses (PRISMA) reporting guideline.

### Data Extraction

Data for each outcome were directly extracted according to the study authors’ outcome definitions and were not modified after extraction. A full list of extracted outcomes and trial characteristics is available in the eMethods in the Supplement. A list of the included studies is available in the eAppendix in the Supplement.

### Statistical Analysis

Meta-analyses of proportions were conducted to pool estimates for CNS objective response rate (ORR) and CNS disease control rate (DCR) reported by more than 5 studies. The random-effects model was used and estimated with the restricted maximum likelihood method. Statistical tests included the *Q* statistic, τ^2^, and *I*^2^.^22^ The full statistical analysis is available in the eMethods in the Supplement. In addition, a comparative meta-analysis was conducted to calculate risk ratios for CNS ORR and CNS DCR by aggregating results from the 2 RCTs.^23,24^ All statistical analyses were conducted using the R programming language (R Foundation).^25,26,27^ The threshold for statistical significance was α = .05. All tests were 2 sided.

### Assessment of Study Quality

Phase 3 trials were assessed using the Cochrane risk of bias tool.^28^ Phase 2 and retrospective trials were assessed using a modified version of the Newcastle-Ottawa Scale for cohort studies.^29^

## Results

### Study Characteristics

**Figure 1.**
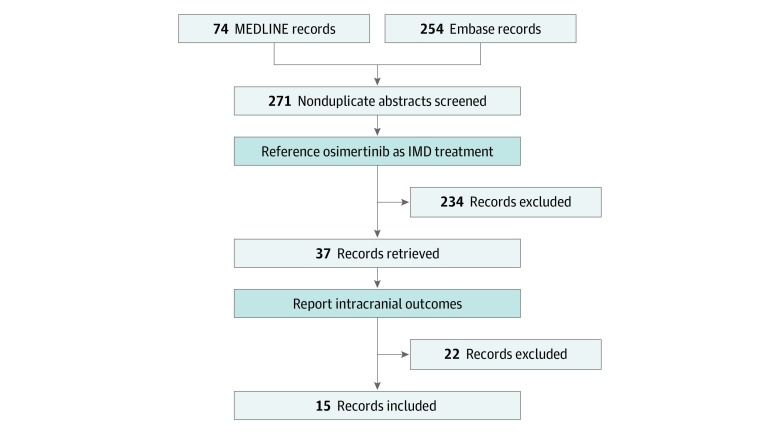
Preferred Reporting Items for Systematic Reviews and Meta-analyses (PRISMA) Flow Diagram^42^ Studies were included that reported intracranial outcomes for osimertinib mesylate in the management of intracranial metastatic disease (IMD). Records were identified from MEDLINE and Embase databases and included published articles and abstracts.

Among 271 records identified in the systematic review, 324 patients with metastatic EGFR-variant NSCLC and IMD receiving osimertinib in 15 studies^19,23,24,30,31,32,33,34,35,36,37,38,39,40,41^ retrieved from the literature search fulfilled eligibility criteria for inclusion in the meta-analysis (Figure 1).^42^ These consisted of 2 RCTs (AURA3^23^ and FLAURA^24^), 1 nonrandomized clinical trial,^30^ four single-arm trials (AURA17,^31^ ASTRIS,^32^ APOLLO,^19^ and a study by Peled et al^33^), 1 pooled analysis of 2 single-arm trials^34^ (AURA extension^43^ and AURA2^44^), 1 retrospective multi-institution single-arm cohort study,^35^ and 6 retrospective single-institution single-arm cohort studies.^36,37,38,39,40,41^ Table 1 summarizes characteristics of the 15 studies.^19,23,24,30,31,32,33,34,35,36,37,38,39,40,41^

**Table 1.** Study Characteristics

Source	Patients, No.	Study phase	Comparators	Trial name	Publication type	Therapy line	Pharmaceutical industry funding
Devjak et al,^40^ 2018	10	Retrospective	NR	NR	Abstract	Any	No
Gadgeel et al,^35^ 2017	45	Retrospective	NR	NR	Abstract	Second	NR
Goss et al,^34^ 2018	50	2	NR	AURA2, AURA extension	Article	Any	Yes
Iuchi et al,^30^ 2018	17	3	Gefinitib, erlotinib hydrochloride, or afatinib dimaleate	NR	Abstract	Second	NR
Kim et al,^32^ 2017	NR	3	NR	ASTRIS	Abstract	Any	Yes
Mu et al,^36^ 2018	15	Retrospective	NR	NR	Abstract	Second	NR
Park et al,^41^ 2018	14	2	NR	NR	Abstract	First	No
Peled et al,^33^ 2018	20	2	NR	NR	Abstract	Any	No
Reungwetwattana et al,^24^ 2018	22	3 (RCT)	Gefitinib or erlotinib hydrochloride	FLAURA	Article	First	Yes
Sonoda et al,^37^ 2017	NR	Retrospective	NR	NR	Abstract	Any	NR
Wu et al,^23^ 2018	30	3 (RCT)	Platinum–pemetrexed disodium	AURA3	Article	Second	Yes
Xie et al,^38^ 2019	31	Retrospective	NR	NR	Article	Second	NR
Xing et al,^19^ 2018	32	2	NR	APOLLO	Abstract	Second	Yes
Xing et al,^39^ 2019	15	Retrospective	NR	NR	Article	Second	No
Zhou et al,^31^ 2017	23	2	NR	AURA17	Abstract	Second	Yes

Abbreviations: APOLLO, Open Label, Prospective Study to Investigate Efficacy and Safety of AZD9291 in BM (brain metastases] From NSCLC [non–small cell lung cancer] Patients With EGFR T790M; ASTRIS, Real World Treatment Study of AZD9291 for Advanced/Metastatic EGFR T790M Mutation NSCLC; AURA, AZD9291 vs Platinum-Based Doublet-Chemotherapy in Locally Advanced or Metastatic Non–Small Cell Lung Cancer; FLAURA, AZD9291 vs Gefitinib or Erlotinib in Patients With Locally Advanced or Metastatic Non–Small Cell Lung Cancer; NR, not reported; RCT, randomized clinical trial.

Data were extracted from published studies and supplements. These data were pooled using a random-effects model. Risk of bias was assessed using the Cochrane risk of bias tool and the modified Newcastle-Ottawa Scale.

### CNS ORR and CNS DCR

**Figure 2.**
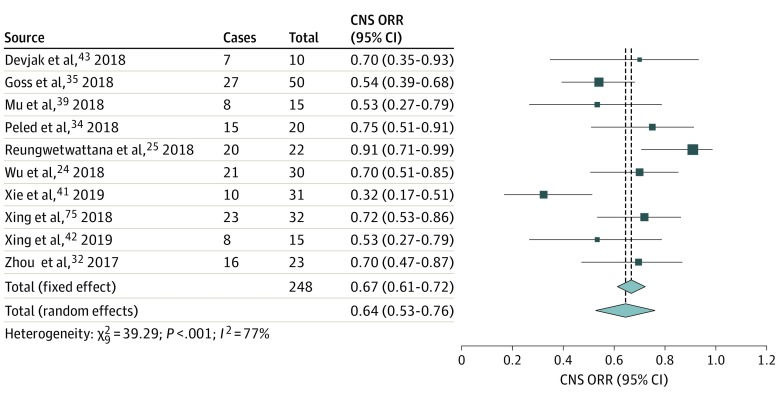
Forest Plot of Central Nervous System (CNS) Objective Response Rate (ORR) The CNS ORRs were either taken directly from individual studies or calculated using reported numbers of responding and total treated patients. The size of each box represents the weight by the random-effects method of the contribution of each study to the weight of the sample in meta-analysis. The vertical dashed lines indicate the point of summary CNS ORRs, and the diamonds indicate the 95% CI for the summary CNS ORRs. Analyses using the inverse variance method were performed with the R programming language^25,26,27^ and the R packages metafor^26^ and meta.^27^

**Figure 3.**
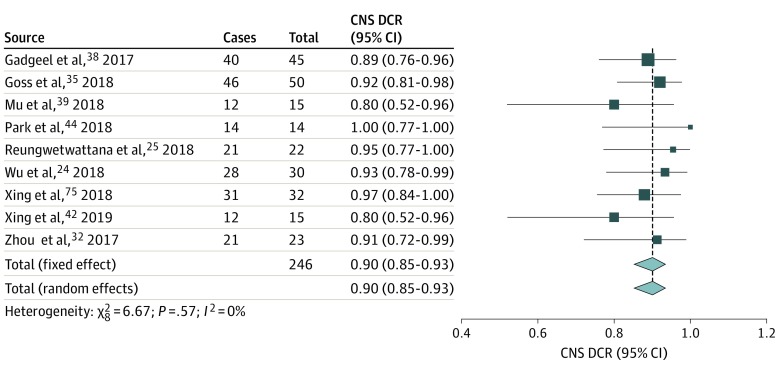
Forest Plot of Central Nervous System (CNS) Disease Control Rate (DCR) The CNS DCRs were either taken directly from individual studies or calculated using reported numbers of responding and total treated patients. The size of each box represents the weight by the random-effects method of the contribution of each study to the weight of the sample in meta-analysis. The vertical dashed line indicates the point of summary CNS DCRs, and the diamonds indicate the 95% CI for the summary CNS DCRs. Analyses using the inverse variance method were performed with the R programming language^25,26,27^ and the R packages metaphor^26^ and meta.^27^

Meta-analyses of proportions generated summary estimates for CNS ORR^19,23,24,31,33,34,36,38,39,40^ of 64% (95% CI, 53%-76%; n = 195) (Figure 2) and for CNS DCR^19,23,24,31,34,35,36,39,41^ of 90% (95% CI, 85%-93%; n = 246) (Figure 3).^25,26,27^ Results of statistical tests indicated high heterogeneity for CNS ORR (*I*^2^ = 77%, 95% CI, 58%-88%; τ^2^ = 0.0268, 95% CI, 0.0054-0.0938; *Q* = 39.29, *P* < .001) and low heterogeneity for CNS DCR (*I*^2^ = 0%, 95% CI, 0%-58%; τ^2^ = 0, 95% CI, 0-1.25; *Q* = 6.67, *P* = .57). Other intracranial outcomes, such as CNS progression-free survival and CNS time to response, were not reported in sufficient numbers or did not include measures of uncertainty to allow for meta-analysis. Twelve studies^9,23,24,31,32,33,34,36,37,38,39,40^ reported CNS ORR, but 2 of these studies^32,37^ were excluded from the primary analysis because they did not report the numbers of patients receiving and responding to osimertinib, which were used to calculate CNS ORR.

A secondary analysis of CNS ORR was prompted by the high heterogeneity of the primary analysis, resulting in a secondary summary estimate for CNS ORR of 65% (95% CI, 65%-72%) (eFigure 1 in the Supplement). From the primary analysis of 10 studies^19,23,24,31,33,34,36,38,39,40^ reporting CNS ORR, 2 studies^24,38^ were identified as outliers visually on a leave-out-1 forest plot (eFigure 2 in the Supplement) and by scores on the *influence* function in the R metafor package.^26^ Funnel plots generated for CNS ORR (eFigure 3 in the Supplement) and CNS DCR (eFigure 4 in the Supplement) failed to show asymmetry that indicated publication bias, consistent with unweighted Egger regression. Subgroup analyses did not reveal additional sources of heterogeneity for CNS ORR or CNS DCR (eFigures 5-14 in the Supplement).

A comparative meta-analysis was conducted to examine CNS ORR and CNS DCR in osimertinib vs comparator using data from the 2 included RCTs.^23,24^ The summary relative risk for an objective CNS response (ie, CNS ORR) was 1.44 (95% CI, 1.06-1.96; *P* = .02) favoring osimertinib vs comparator (eFigure 15 in the Supplement). The summary relative risk for CNS disease control (ie, CNS DCR) was 1.13 (95% CI, 0.96-1.33; *P* = .14) favoring osimertinib vs comparator, although the result was not statistically significant (eFigure 16 in the Supplement).

### Other Effectiveness Outcomes

The median CNS progression-free survival was reported in 2 studies^19,23^ as 10.9 (95% CI, 6.1 to not reached) months and 11.7 (95% CI, 10 to not reached) months, respectively, with 7 other studies^24,31,32,33,34,36,39^ reporting that the median CNS progression-free survival was not reached. The median CNS duration of response ranged from 8.9 to 15.2 months in 5 studies,^19,23,24,30,31^ with 1 reported lower confidence bound at 4.1 months. The CNS time to response was reported in 4 studies,^23,24,36,39^ ranging from 1.3 to 1.5 months. The median best change in intracranial lesion size ranged from −40% to −64% in 5 studies,^23,24,34,36,39^ with complete intracranial response rates of 7% to 23% in 6 studies.^23,24,34,36,38,39^ The median overall survival was reported in only 1 study,^38^ with a value of 16.2 months. Three studies^35,39,41^ reported that the median overall survival was not reached before data cutoff. The median follow-up length ranged in 9 studies^23,24,31,33,34,35,36,38,39^ from 5.5 to 12.4 months, and follow-up completeness was reported in 2 studies^32,38^ at 78% and 86%. Table 2 summarizes extracted outcome data in the 15 studies.^19,23,24,30,31,32,33,34,35,36,37,38,39,40,41^

**Table 2.** Summary of Extracted Outcome Data

Source	OS	CNS DCR, median (IQR), %	CNS ORR, %	CNS PFS (cEFR), median, mo	CNS PFS (cFAS), median (IQR), mo	CNS DoR, median (IQR), mo	CNS TTR, median, mo	Best change in intracranial lesion size, median%	Complete response rate, %	Follow-up length, median, mo	CTCAE grade ≥3 adverse event rate, %
Devjak et al,^40^ 2018	NR	NR	70	NR	NR	NR	NR	NR	NR	NR	NR
Gadgeel et al,^35^ 2017	70% At 1 y	88	NR	NR	NR	NR	NR	NR	NR	7.1	NR
Goss et al,^34^ 2018	NR	92 (81-98)	54	Not reached (7 to not reached)	NR	Not reached	NR	−53	12	11	38 (cEFR)
Iuchi et al,^30^ 2018	NR	NR	NR	NR	NR	13.8	NR	NR	NR	NR	NR
Kim et al,^32^ 2017	NR	NR	81.3	Not reached	Not reached	Not reached	NR	NR	NR	NR	NR
Mu et al,^36^ 2018	NR	80	53.3	Not reached	NR	NR	1.3	−40	23	6.5	NR
Park et al,^41^ 2018	Not reached	100	NR	Not reached	Not reached	NR	NR	NR	NR	NR	NR
Peled et al,^33^ 2018	NR	NR	75	Not reached	NR	NR	NR	NR	NR	10	NR
Reungwetwattana et al,^24^ 2018	NR	95 (77-100)	91	NR	Not reached (16.5 to not reached)	15.2 (4.1 To not reached)	1.5	−64	23	12.4	33
Sonoda et al,^37^ 2017	NR	NR	67	NR	NR	NR	NR	NR	NR	NR	30
Wu et al,^23^ 2018	NR	93 (81-98)	70	NR	11.7 (10 To not reached)	8.9 (4.3 To not reached)	1.5	−43	7	5.5	19
Xie et al,^38^ 2019	16.2 mo	NR	32.3	NR	NR	NR	NR	NR	10	8.5	NR
Xing et al,^19^ 2018	NR	97	71.9	10.9 (6.1 To not reached)	NR	8.3 (5.8 To not reached)	NR	NR	NR	NR	39.5
Xing et al,^39^ 2019	Not reached	80 (57-100)	53.3	Not reached	NR	NR	1.3	−40	20	6.5	22.7
Zhou et al,^31^ 2017	NR	91 (73-93)	70	Not reached (9.4 to not reached)	Not reached	11.1 (8.2 To not reached)	NR	NR	NR	8.2	NR

Abbreviations: cEFR, central nervous system evaluable for response set; cFAS, central nervous system full analysis set; CNS, central nervous system; CTCAE, Common Terminology Criteria for Adverse Events (version 3.0); DCR, disease control rate; DoR, duration of response; IQR, interquartile range; NR, not reported; ORR, objective response rate; OS, overall survival; PFS, progression-free survival; TTR, time to response.

### Safety

Of the 15 included studies,^19,23,24,30,31,32,33,34,35,36,37,38,39,40,41^ safety outcomes were reported in 7 studies.^19,23,24,34,37,38,39^ In their retrospective study of 40 patients with IMD receiving osimertinib, Xie et al^38^ reported that 1 patient experienced toxic effects, resulting in fatal pneumonitis. Xing et al^39^ reported that of 22 patients receiving osimertinib 5 had adverse events of Common Terminology Criteria for Adverse Events (version 3.0) (CTCAE) grade 3 or higher, including 2 with anorexia, 1 with stomatitis, 1 with fatigue, and 1 with decreased platelet count. Sonoda et al^37^ reported that among 30 patients receiving osimertinib the most frequent adverse events of CTCAE grade 3 or higher were rash in 13% of patients, pneumonitis in 13%, and neutropenia in 7%. Wu et al^23^ and Reungwetwattana et al^24^ reported that of 75 patients and 61 patients, respectively, receiving osimertinib in their CNS full analysis cohorts, 3 patients and 10 patients, respectively, experienced adverse events of CTCAE grade 3 or higher that were possibly associated with treatment. In their study of 50 patients receiving osimertinib, Goss et al^34^ reported that 6 patients in the CNS evaluable for response set (cEFR) group had adverse events of CTCAE grade 3 or higher that were possibly associated with treatment, with 1 case of fatal interstitial lung disease. They reported that every patient in the cEFR group experienced at least 1 adverse event of any CTCAE grade.^34^ Central nervous system toxic effects were reported only in the study of 22 patients receiving osimertinib by Xing et al,^39^ with 1 patient experiencing CTCAE grade 3 fatigue and more patients experiencing CTCAE grade 1 or 2 adverse events (7 with fatigue, 4 with nausea, 3 with vomiting, 2 with headache, and 3 with dizziness). The authors of other studies^23,24,34,39^ stated that their safety results for patients with IMD receiving osimertinib were consistent with results in their overall patient populations.

### Assessment of Study Quality

Results from the evaluation of study quality are shown in eFigure 17 and eFigure 18 in the Supplement. Only 1 study^24^ reported blinding of participants and personnel, and only 2 studies^32,38^ reported the percentage of patients lost to follow-up. Six studies^19,23,24,31,32,34^ reported pharmaceutical industry funding. Overall, 6 studies^19,23,32,37,38,40^ were at high risk of bias in 1 criterion, and 3 studies^30,31,34^ were at high risk of bias in 2 criteria.

## Discussion

### Effectiveness

The CNS ORR reported herein (64%; 95% CI, 53%-76%) confirms and strengthens results reported in a pooled analysis of the CNS data from the AURA2 and AURA extension trials (54%; 95% CI, 39%-68%; n = 50).^34^ However, 2 outlier studies^24,38^ excluded from the final meta-analysis of CNS ORRs reported values more than 2 SDs from the summary effect size. The first outlier study^38^ examined osimertinib effectiveness in 40 patients with IMD, grouped as having either progressive untreated CNS disease, progressive radiotherapy-treated CNS disease, or stable CNS disease. The CNS ORR reported in that study (32% [10 of 31 patients]) may be lower than the value reported herein because of the inclusion of patients with progressive IMD, which was an exclusion criterion in other studies included in the present analysis. The second outlier study^24^ reported a CNS ORR of 91% (95% CI, 71%-99%; 20 of 22 patients). This percentage may be higher than the value reported herein because the patients in that study had not received any previous EGFR-TKI treatment, in contrast to the patients in many of the included studies^19,23,31,33,34,36,38,39,40^ who received osimertinib as second-line or third-line therapy.

The CNS ORR reported herein is also consistent with the CNS effectiveness reported for other BBB-penetrant targeted therapies for NSCLC. One analysis of the CNS data for patients with measurable IMD who received alectinib hydrochloride for anaplastic lymphoma kinase (ALK)–positive NSCLC reported a CNS ORR of 64.0% (95% CI, 49.2%-77.1%; 32 of 50 patients).^45^ Another study^46^ in patients receiving brigatinib for ALK-positive NSCLC reported a CNS ORR of 67% (95% CI, 41%-87%; 12 of 18 patients). Similar values for CNS ORR have been reported for crizotinib, ceritinib, and lorlatinib.^47^ In comparison, CNS ORRs for less BBB-penetrant targeted therapies for patients with NSCLC and IMD have been reported at lower values for ceritinib (45.0%; 95% CI, 23.1%-68.5%; 9 of 20 patients), an ALK inhibitor,^48^ and for erlotinib hydrochloride (44.3%; 95% CI, 35.8%-53.1%; n = 238), an EGFR inhibitor.^49^ It is possible that these discrepancies are because of differences in sample size.

The CNS DCR was reported herein to be 90% (95% CI, 85%-93%). This percentage is higher than values reported for crizotinib at 12 weeks among previously treated patients^50^ (62%; 95% CI, 54%-70%; n = 166) and values reported in an analysis of 16 studies^49^ for gefitinib and erlotinib (75.7%; 95% CI, 70.3%-80.5%; n = 434) but is consistent with values reported for ceritinib and alectinib.^20,49,50^ In both CNS ORR and CNS DCR, the Egger regression test failed to indicate publication bias, but this test may have been underpowered.

A comparative analysis was conducted to assess the risk ratios for CNS ORR and CNS DCR among the 2 included RCTs.^23,24^ These results may lend accuracy to estimates of effectiveness for osimertinib vs other therapies, although the validity of this analysis is limited by the lack of statistical significance of the result for CNS DCR and the difference in comparator groups between studies.

Reporting of additional outcomes was inconsistent among included studies. The median CNS progression-free survival with osimertinib was reported by Xing et al^19^ as 10.9 (95% CI, 6.1 to not reached; n = 32) months in their cEFR group. This result is consistent with values for the median CNS progression-free survival reported for bevacizumab (7.8; 95% CI, 7.1-8.5 months; n = 51), erlotinib (10.1; 95% CI, 7.1-12.3 months; n = 48), and icotinib (10.0; 95% CI, 5.6-14.4 months; n = 85), for example.^51,52,53^ The intracranial effectiveness of other TKIs is further addressed in a recent review article.^54^

### Safety

Prevalence of adverse events CTCAE grade 3 or higher ranged from 19% to 39% in the present study. Two instances of fatal toxic effects among 149 patients receiving osimertinib were reported, including 1 case of pneumonitis^38^ and 1 case of interstitial lung disease.^34^ In comparison, CTCAE grade 3 or higher adverse event rates have been reported to be 84% for bevacizumab plus paclitaxel plus carboplatin, 54% for bevacizumab plus erlotinib, 45% for erlotinib, 29% for gefitinib, 65% for ceritinib, 41% for alectinib, and 36% for afatinib dimaleate.^20,55^ Most included studies^23,24,34,37,39^ herein reported that 100% or near 100% of patients receiving osimertinib experienced at least 1 adverse event of any CTCAE grade, although rates of possibly associated adverse events of CTCAE grade 3 or higher were reported at 4% to 12%.^23,34^

### Limitations

This study has several limitations. First, effectiveness measures reported herein only provide snapshots of patient survival. Overall survival is the criterion-standard outcome for assessing survival benefit and was infrequently reported among included studies, which may be because of the inclusion and exclusion criteria of our study. However, CNS progression-free survival data were also largely unreported, possibly owing to short study length. Increased reporting of key survival outcomes and complete safety data would further contextualize reported effectiveness.

Second, meta-analyses of single-arm studies are noncomparative in nature. The noncomparative results of this study do not support the use of any single therapy over another and only lend precision to existing descriptive results. The comparative results of our study are based on end points that are dependent on tumor assessment, which require confirmatory data.

Third, the definition of *intracranial response* differed between included studies. Of the 12 total studies^19,23,24,31,32,33,34,36,37,38,39,40^ reporting CNS ORR, 9 studies^23,24,31,32,34,36,37,39,40^ defined treatment response according to Response Evaluation Criteria in Solid Tumors (RECIST; version 1.1),^56^ 1 study^38^ was based on a modified RECIST 1.1, and 2 studies^19,33^ did not report definitions of intracranial response. However, RECIST 1.1 is only applicable to tumors at least 10 mm in their longest dimension and may not account for responses in smaller tumors. In addition, few studies^23,24,34,36,38,39^ reported on complete and partial intracranial response rates, which would lend precision to an estimate of intracranial effectiveness and facilitate comparison between studies.

Fourth, although this study is limited to IMD from NSCLC, EGFR alterations are present in other cancers, including head and neck squamous cell carcinomas, anal squamous cell carcinomas, and gliomas.^57,58^ Future trials may support a role for osimertinib in IMD (or primary disease in the case of glioma) from these diseases, and future meta-analysis may examine the role of osimertinib in IMD from a larger patient population stratified by primary disease type.

### Future Directions

Ongoing and recent larger trials may refine the estimates for intracranial effectiveness and safety of osimertinib. The ASTRIS trial is a global study of 3015 patients who received osimertinib in a real-world setting.^18^ Data from that trial may identify factors associated with therapeutic response. An osimertinib trial specifically for patients with IMD is ongoing.^59^ Together, studies like these may help progress IMD management in the era of precision medicine.^33^

Central nervous system effectiveness should remain a target of future therapeutic development strategies. Novel targeted therapies have demonstrated preclinical CNS results that may support a role in the treatment or prevention of IMD, with improved selectivity for EGFR alterations and reduced CNS efflux compared with osimertinib.^60,61^ In addition, innovation in therapeutic delivery modalities may guide treatment sequencing or support the use of existing systemic treatments, which have historically been limited by their lack of BBB permeability. Methods of increasing BBB penetrance of systemic drugs include modification through rational drug design, conjugation to ligands targeted to receptor-mediated transport, and disruption of the BBB through the use of osmotic media, biochemical agents, focused ultrasonography, or radiotherapy.^62,63^ A role for osimertinib and other targeted therapies may involve auxiliary therapies or delivery methods like these. Future trials will also need to consider the combination of osimertinib with other modalities, such as neurosurgery and radiotherapy, to clarify the suitability of osimertinib for patients with IMD as either adjunct therapy or monotherapy.

## Conclusions

These results support a potential role for osimertinib in the treatment of patients with IMD, but it is unclear whether that may be as an adjunct therapy or a nonadjuvant therapy or as a replacement for standard frontline therapies, such as neurosurgery or radiotherapy. Trials in oncology should continue to include patients with IMD to clarify the use of novel therapies for these individuals. Advances in tumor genotyping and subgroup analyses from large trials may better assess responses to targeted therapies on an individual patient basis and more precisely define the role of osimertinib and other targeted therapies in IMD management.

## Notes

Supplement. **eMethods.** Supplementary Methods **eAppendix. **List of Included Studies **eFigure 1.** Initial Meta-analysis of CNS Objective Response Rate (ORR) Prior to Sensitivity Analysis **eFigure 2.** Leave-Out-One Sensitivity Analysis for Identifying Outlier Studies for CNS Objective Response Rate (ORR) **eFigure 3.** Funnel Plot for Publication Bias in CNS Objective Response Rate (ORR) **eFigure 4.** Funnel Plot for Publication Bias in CNS Disease Control Rate (DCR) **eFigure 5.** Forest Plot of Reported CNS ORR, Stratified by Retrospective Versus Prospective Studies **eFigure 6.** Forest Plot of Reported CNS ORR, Stratified by Line of Therapy **eFigure 7.** Forest Plot of Reported CNS ORR, Stratified by Pharmaceutical Industry Funding **eFigure 8.** Forest Plot of Reported CNS ORR, Stratified by Randomized Controlled Trials Versus Other Study Types **eFigure 9.** Forest Plot of Reported CNS ORR, Stratified by Abstract Versus Article **eFigure 10.** Forest Plot of Reported CNS DCR, Stratified by Retrospective Versus Prospective **eFigure 11.** Forest Plot of Reported CNS DCR, Stratified by Line of Therapy **eFigure 12.** Forest Plot of Reported CNS DCR, Stratified by Pharmaceutical Industry Funding **eFigure 13.** Forest Plot of Reported CNS DCR, Stratified by Randomized Controlled Trials Versus Other Study Types **eFigure 14.** Forest Plot of Reported CNS DCR, Stratified by Abstract Versus Article **eFigure 15.** Forest Plot of Risk Ratio for CNS ORR Among Included Randomized Controlled Trials **eFigure 16.** Forest Plot of Risk Ratio for CNS DCR Among Included Randomized Controlled Trials **eFigure 17.** Cochrane Risk of Bias Tool Assessment of Phase III Studies **eFigure 18.** Modified Newcastle-Ottawa Scale Assessment of Phase II and Retrospective Studies **eReferences.**
