# Supplementary material for: Assessment of Effectiveness and Safety of Osimertinib for Patients With Intracranial Metastatic Disease: A Systematic Review and Meta-analysis
Source: JAMA Netw Open. 2020 Mar 25;3(3):e201617. doi: 10.1001/jamanetworkopen.2020.1617 (PMC7097701; doi:10.1001/jamanetworkopen.2020.1617)
Supplement: Supplement. — eMethods. Supplementary Methods eAppendix. List of Included Studies eFigure 1. Initial Meta-analysis of CNS Objective Response Rate (ORR) Prior to Sensitivity Analysis eFigure 2. Leave-Out-One Sensitivity Analysis for Identifying Outlier Studies for CNS Objective Response Rate (ORR) eFigure 3. Funnel Plot for Publication Bias in CNS Objective Response Rate (ORR) eFigure 4. Funnel Plot for Publication Bias in CNS Disease Control Rate (DCR) eFigure 5. Forest Plot of Reported CNS ORR, Stratified by Retrospective Versus Prospective Studies eFigure 6. Forest Plot of Reported CNS ORR, Stratified by Line of Therapy eFigure 7. Forest Plot of Reported CNS ORR, Stratified by Pharmaceutical Industry Funding eFigure 8. Forest Plot of Reported CNS ORR, Stratified by Randomized Controlled Trials Versus Other Study Types eFigure 9. Forest Plot of Reported CNS ORR, Stratified by Abstract Versus Article eFigure 10. Forest Plot of Reported CNS DCR, Stratified by Retrospective Versus Prospective eFigure 11. Forest Plot of Reported CNS DCR, Stratified by Line of Therapy eFigure 12. Forest Plot of Reported CNS DCR, Stratified by Pharmaceutical Industry Funding eFigure 13. Forest Plot of Reported CNS DCR, Stratified by Randomized Controlled Trials Versus Other Study Types eFigure 14. Forest Plot of Reported CNS DCR, Stratified by Abstract Versus Article eFigure 15. Forest Plot of Risk Ratio for CNS ORR Among Included Randomized Controlled Trials eFigure 16. Forest Plot of Risk Ratio for CNS DCR Among Included Randomized Controlled Trials eFigure 17. Cochrane Risk of Bias Tool Assessment of Phase III Studies eFigure 18. Modified Newcastle-Ottawa Scale Assessment of Phase II and Retrospective Studies eReferences. [file jamanetwopen-3-e201617-s001.pdf]

Supplementary Online Content

Erickson AW, Brastianos PK, Das S. Assessment of effectiveness and safety of osimertinib for patients with intracranial metastatic disease: a systematic review and meta-analysis. *JAMA Netw Open*. 2020;3(3):e201617. doi:10.1001/jamanetworkopen.2020.1617

eMethods. Supplementary Methods
eAppendix. List of Included Studies
eFigure 1. Initial Meta-analysis of CNS Objective Response Rate (ORR) Prior to Sensitivity Analysis
eFigure 2. Leave-Out-One Sensitivity Analysis for Identifying Outlier Studies for CNS Objective Response Rate (ORR)
eFigure 3. Funnel Plot for Publication Bias in CNS Objective Response Rate (ORR)
eFigure 4. Funnel Plot for Publication Bias in CNS Disease Control Rate (DCR)
eFigure 5. Forest Plot of Reported CNS ORR, Stratified by Retrospective Versus Prospective Studies
eFigure 6. Forest Plot of Reported CNS ORR, Stratified by Line of Therapy
eFigure 7. Forest Plot of Reported CNS ORR, Stratified by Pharmaceutical Industry Funding
eFigure 8. Forest Plot of Reported CNS ORR, Stratified by Randomized Controlled Trials Versus Other Study Types
eFigure 9. Forest Plot of Reported CNS ORR, Stratified by Abstract Versus Article
eFigure 10. Forest Plot of Reported CNS DCR, Stratified by Retrospective Versus Prospective
eFigure 11. Forest Plot of Reported CNS DCR, Stratified by Line of Therapy
eFigure 12. Forest Plot of Reported CNS DCR, Stratified by Pharmaceutical Industry Funding
eFigure 13. Forest Plot of Reported CNS DCR, Stratified by Randomized Controlled Trials Versus Other Study Types
eFigure 14. Forest Plot of Reported CNS DCR, Stratified by Abstract Versus Article
eFigure 15. Forest Plot of Risk Ratio for CNS ORR Among Included Randomized Controlled Trials
eFigure 16. Forest Plot of Risk Ratio for CNS DCR Among Included Randomized Controlled Trials
eFigure 17. Cochrane Risk of Bias Tool Assessment of Phase III Studies
eFigure 18. Modified Newcastle-Ottawa Scale Assessment of Phase II and Retrospective Studies
eReferences.

This supplementary material has been provided by the authors to give readers additional information about their work.

© 2020 Erickson AW et al. *JAMA Network Open*.

eMethods. Supplementary Methods

Search and selection criteria

A literature search was conducted on September 20, 2019, using the following search query in MEDLINE and EMBASE databases: (osimertinib OR mereletinib OR tagrisso OR tamarix OR azd9291) AND (brain metastases OR intracranial metastatic disease OR cns). Only articles and abstracts published in English were considered, and all years considered from database inception to search date. Study authors were not contacted. Retrieved records were screened by abstract for reference to osimertinib as treatment for IMD. Case reports, case series, and reviews were excluded. Records reporting intracranial outcomes were included in the analysis.

Data extraction

The following outcomes were extracted from included studies: CNS objective response rate (ORR), CNS disease control rate (DCR), CNS progression-free survival (PFS), CNS duration of response (DoR), CNS time to response (TTR), best change in intracranial lesion size, complete response rate, overall survival (OS), follow-up length, and follow-up completeness. Data for each outcome were directly extracted according to the original authors' outcome definitions, and not modified following extraction. Where available, results were extracted from data from specified subgroups of patients whose CNS disease was evaluable for response (cEFR). Safety data were extracted for adverse events grade 3 or higher according to the Common Terminology Criteria for Adverse Events (CTCAE). Additionally, the following trial characteristics were extracted: author, year, number of patients, study phase, trial name, publication type, therapy line, and pharmaceutical industry funding.

© 2020 Erickson AW et al. *JAMA Network Open*.

Statistical analysis

Meta-analyses of proportions were conducted to pool estimates for outcomes reported by more than five studies, which included CNS ORR and CNS DCR, reported effectively in ten and nine of the included fifteen studies, respectively. The random-effects model was used for weighted synthesis on the assumption that the included studies represented a sample of studies whose true effect sizes followed a normal distribution. This model was estimated using the restricted maximum likelihood method. Statistical tests included the Q-statistic for heterogeneity, τ^2 for between-study variance, and I^2 statistic developed by Higgins et al. as a "signal-to-noise ratio" to assess the percentage of observed variance attributable to variance between studies.[1,2] An I^2 value of 0% was interpreted to indicate all heterogeneity was due to sampling error, and 100% to indicate all variance was due to true differences between studies, with 0–50% as a benchmark for low heterogeneity and 50–100% for high heterogeneity. Initial synthesis of values for CNS ORR generated an I^2 value indicating high heterogeneity. This prompted leave-out-one and influence analyses to identify outlier studies, which were removed to produce the final model. Subgroup analyses were conducted to reveal sources of additional heterogeneity by stratifying studies based on trial characteristics as: retrospective vs. prospective; article vs. abstract; first vs. second vs. any line osimertinib therapy; and pharmaceutical industry funding yes vs. no vs. not reported. Funnel plots were generated to assess for publication bias and unweighted Egger's regression tests performed to assess funnel plot asymmetry. Additionally, a comparative meta-analysis was conducted to calculate risk ratios for CNS ORR and CNS DCR by aggregating results from the two RCTs.[3,4]

© 2020 Erickson AW et al. *JAMA Network Open*.

All statistical analyses were conducted using the R programming language (v3.6.1; R Core Team, 2019) and the R packages *meta* (v4.9-6; Schwarzer, 2019) and *metafor* (v2.1-0; Viechtbauer, 2019).[5-7]

Assessment of study quality

Phase III trials were assessed using the Cochrane Risk of Bias Tool (RoB 2).[8] Phase II and retrospective trials were assessed using a modified version of the Newcastle-Ottawa scale for cohort studies.[9] To modify the scale, three questions were removed—two relating to selection and comparison of non-exposed patients and one question assessing presence of outcome at study start—and one question was added addressing presence of pharmaceutical industry funding. A "truly representative" exposed cohort was defined as a total group of patients who all have IMD and are receiving osimertinib. A "somewhat representative" exposed cohort was defined as a total group of patients where either 1) at least 50% had IMD but all were receiving osimertinib, or 2) all patients had IMD and at least 50% were receiving osimertinib. Adequate follow-up length was considered to be 6 months.

Ethical review of study

This study was not reviewed by any ethics review board as it did not contain live subjects, nor samples, nor individual-level patient data.

© 2020 Erickson AW et al. *JAMA Network Open*.

eAppendix. List of included studies.

Devjak et al.[10]

Gadgeel et al.[11]

Goss et al.[12]

Iuchi et al.[13]

Kim et al.[14]

Mu et al.[15]

Park et al.[16]

Peled et al.[17]

Reungwetwattana et al.[4]

Sonoda et al.[18]

Wu et al.[3]

Xie et al.[19]

Xing, L et al.[20]

Xing, P et al.[21]

Zhou et al.[22]

© 2020 Erickson AW et al. *JAMA Network Open*.

eFigure 1. Forest plot of CNS objective response rate (ORR) following outlier removal.

Two studies were removed from the forest plot in Figure 2 due to severe heterogeneity indicated by the I^2 value to produce this secondary analysis: Xie et al.[19] and Reungwetwattana et al.[4]

Study	Cases	Total	CNS ORR	95% CI
Devjak 2018	7	10	0.70	[0.35; 0.93]
Goss 2018	27	50	0.54	[0.39; 0.68]
Mu 2017	8	15	0.53	[0.27; 0.79]
Peled 2018	15	20	0.75	[0.51; 0.91]
Wu 2018	21	30	0.70	[0.51; 0.85]
Xing, L 2018	23	32	0.72	[0.53; 0.86]
Xing, P 2019	8	15	0.53	[0.27; 0.79]
Zhou 2017	16	23	0.70	[0.47; 0.87]
Total (fixed effect)		195	0.65	[0.58; 0.72]
Total (random effects)			0.65	[0.58; 0.72]

Heterogeneity: $\chi^2_7 = 6.59$ (P = .47), $I^2 = 0\%$

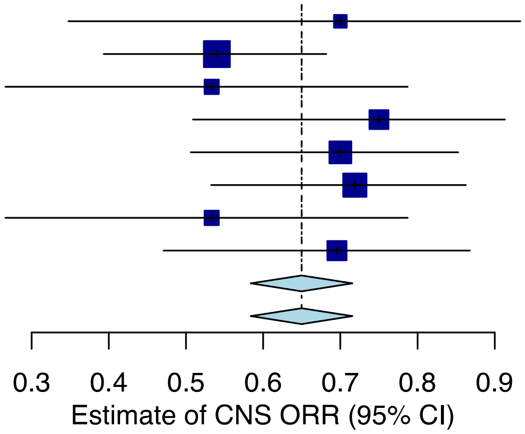

© 2020 Erickson AW et al. *JAMA Network Open*.

eFigure 2. Leave-out-one sensitivity analysis for identifying outlier studies for CNS objective response rate (ORR). A leave-one-out sensitivity analysis calculated summary effect sizes aggregating CNS ORR values from all studies minus one to assess the degree of heterogeneity introduced by any given study. Reungwetwattana et al.[4] and Xie et al.[19] were identified as potential outliers.

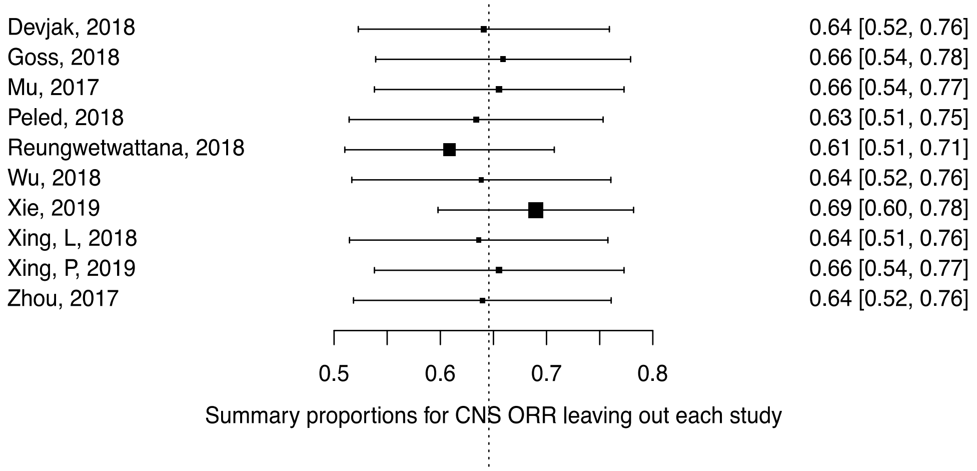

© 2020 Erickson AW et al. *JAMA Network Open*.

eFigure 3. Funnel plot for publication bias in CNS objective response rate (ORR).

Publication bias was not detected on visual inspection of the funnel plot, nor on unweighted Egger's regression test (z = -0.68, p = 0.49).

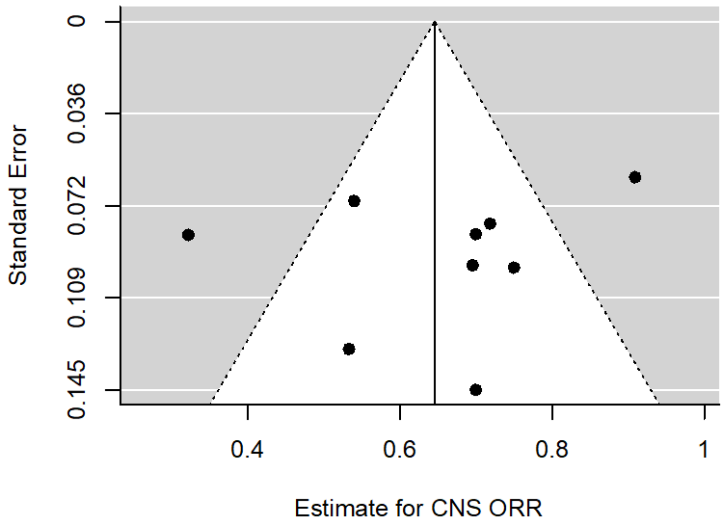

© 2020 Erickson AW et al. *JAMA Network Open*.

eFigure 4. Funnel plot for publication bias in CNS disease control rate (DCR). Publication bias was not detected on visual inspection of the funnel plot, nor on unweighted Egger's regression test (and z = 1.43, p = 0.15).

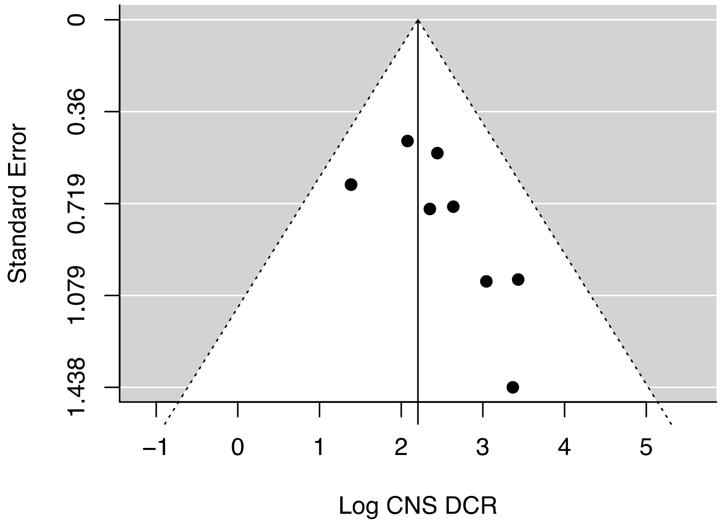

© 2020 Erickson AW et al. *JAMA Network Open*.

eFigure 5. Forest plot of reported CNS ORR, stratified by retrospective versus prospective studies.

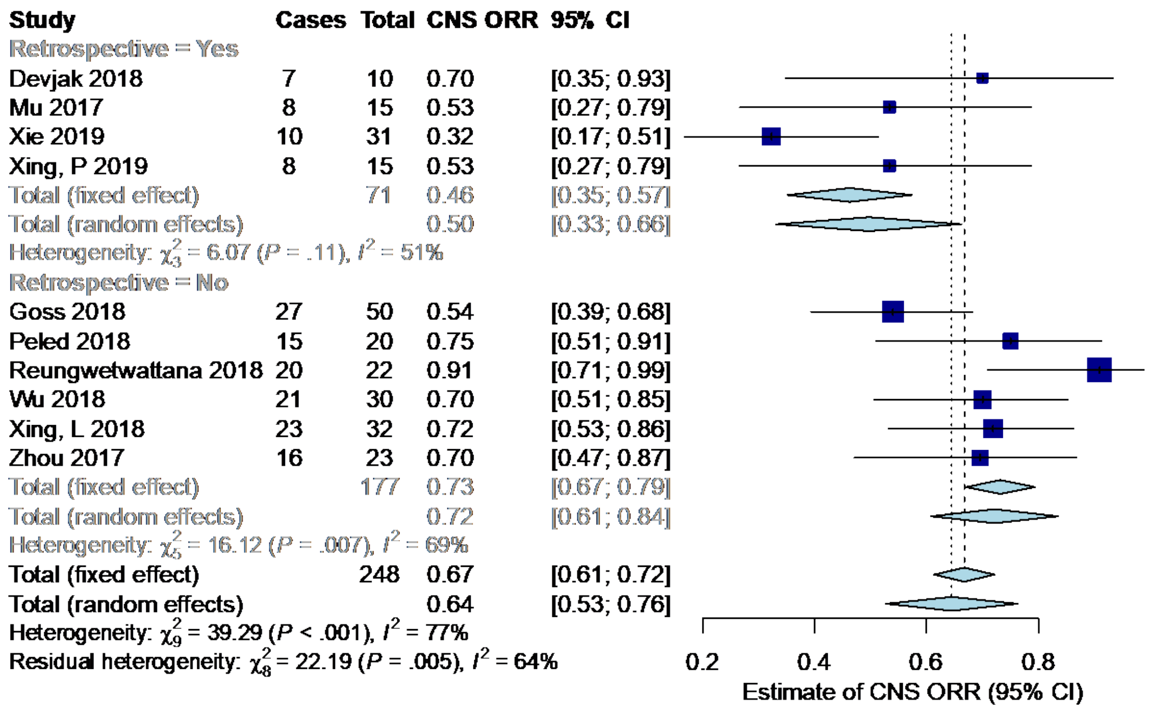

© 2020 Erickson AW et al. *JAMA Network Open*.

eFigure 6. Forest plot of reported CNS ORR, stratified by line of therapy.

Study	Cases	Total	CNS ORR	95% CI
Therapy line = Any				
Devjak 2018	7	10	0.70	[0.35; 0.93]
Goss 2018	27	50	0.54	[0.39; 0.68]
Peled 2018	15	20	0.75	[0.51; 0.91]
Total (fixed effect)		80	0.62	[0.52; 0.73]
Total (random effects)			0.64	[0.50; 0.79]
Heterogeneity: $\chi^2_2 = 3.39$ ($P = .18$), $I^2 = 41\%$				
Therapy line = First				
Reungwetwattana 2018	20	22	0.91	[0.71; 0.99]
Total (fixed effect)		22	0.91	[0.79; 1.00]
Total (random effects)			0.91	[0.79; 1.00]
Heterogeneity: not applicable				
Therapy line = Second				
Mu 2017	8	15	0.53	[0.27; 0.79]
Wu 2018	21	30	0.70	[0.51; 0.85]
Xie 2019	10	31	0.32	[0.17; 0.51]
Xing, L 2018	23	32	0.72	[0.53; 0.86]
Xing, P 2019	8	15	0.53	[0.27; 0.79]
Zhou 2017	16	23	0.70	[0.47; 0.87]
Total (fixed effect)		146	0.59	[0.52; 0.67]
Total (random effects)			0.59	[0.45; 0.73]
Heterogeneity: $\chi^2_5 = 16.09$ ($P = .007$), $I^2 = 69\%$				
Total (fixed effect)		248	0.67	[0.61; 0.72]
Total (random effects)			0.64	[0.53; 0.76]
Heterogeneity: $\chi^2_9 = 39.29$ ($P < .001$), $I^2 = 77\%$				
Residual heterogeneity: $\chi^2_7 = 19.47$ ($P = .007$), $I^2 = 64\%$				

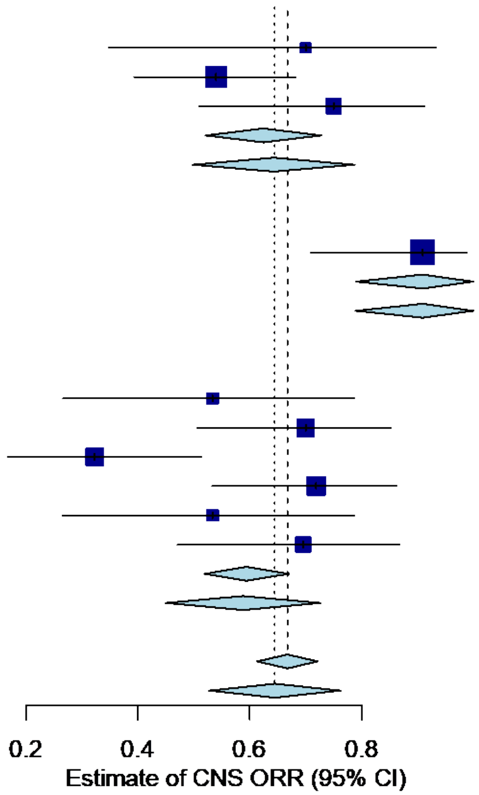

© 2020 Erickson AW et al. *JAMA Network Open*.

eFigure 7. Forest plot of reported CNS ORR, stratified by pharmaceutical industry funding.

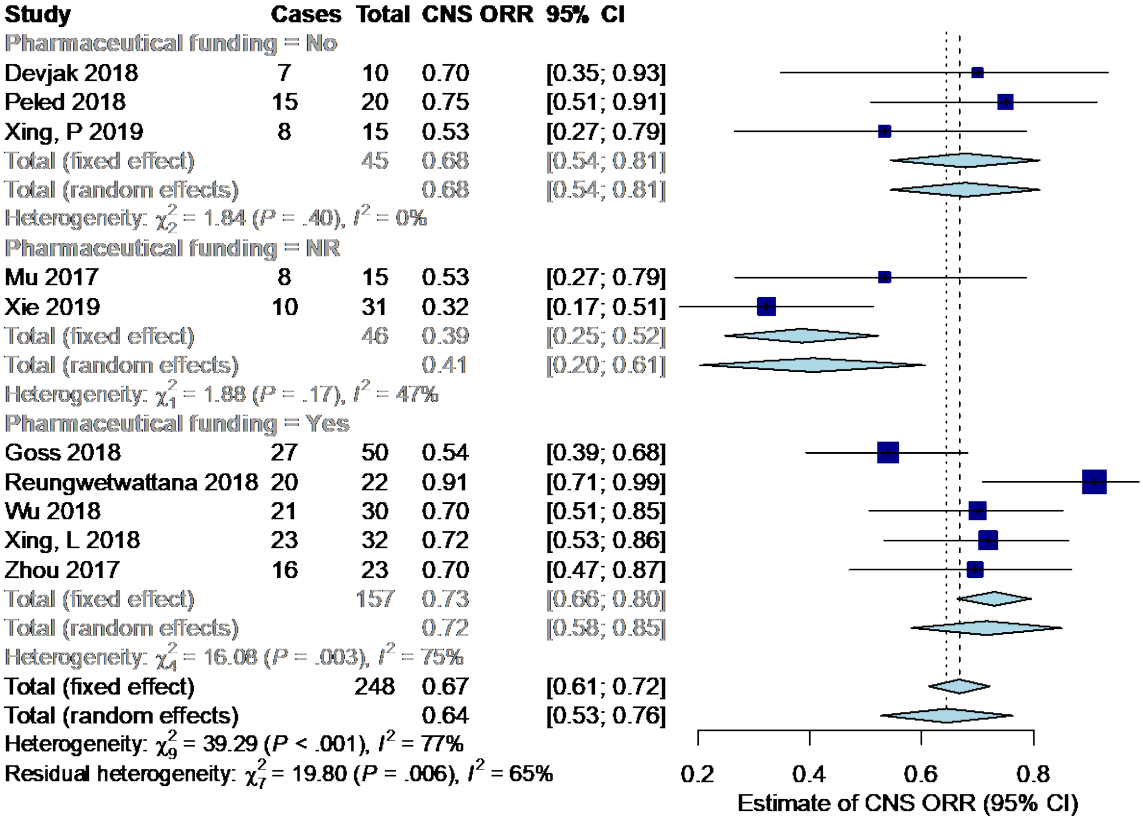

© 2020 Erickson AW et al. *JAMA Network Open*.

eFigure 8. Forest plot of reported CNS ORR, stratified by randomized controlled trials versus other study types.

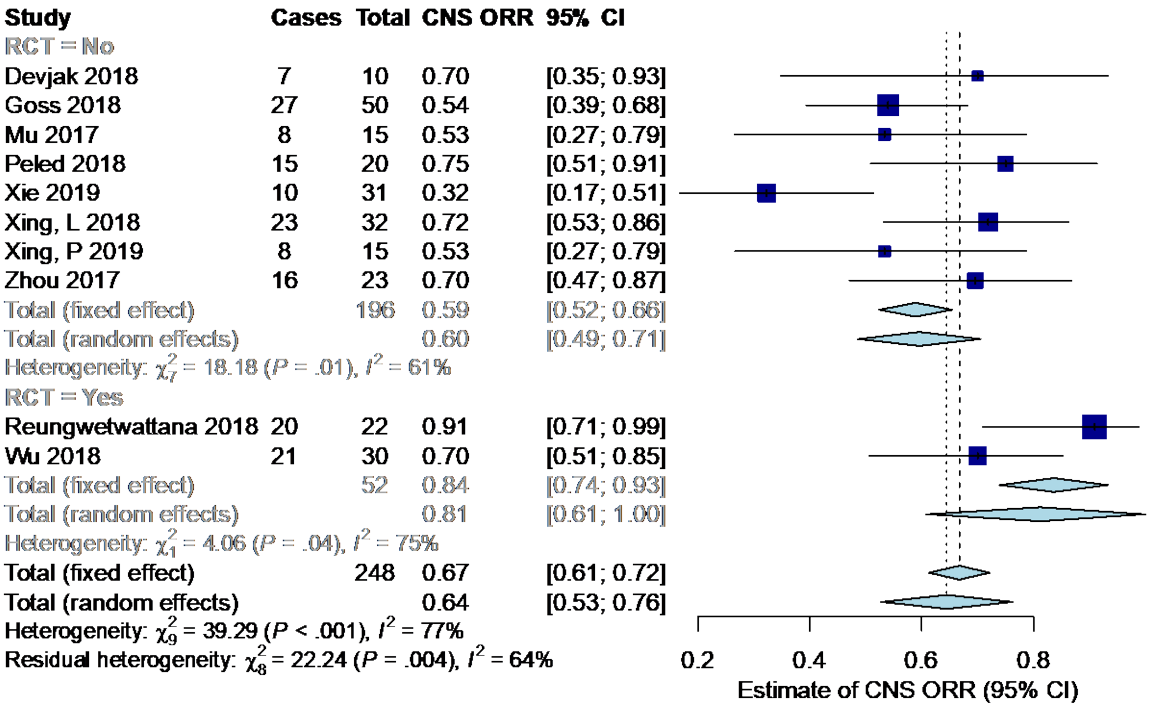

© 2020 Erickson AW et al. *JAMA Network Open*.

eFigure 9. Forest plot of reported CNS ORR, stratified by abstract versus article.

Study	Cases	Total	CNS ORR	95% CI
Publication type = abstract				
Devjak 2018	7	10	0.70	[0.35; 0.93]
Mu 2017	8	15	0.53	[0.27; 0.79]
Peled 2018	15	20	0.75	[0.51; 0.91]
Xing, L 2018	23	32	0.72	[0.53; 0.86]
Zhou 2017	16	23	0.70	[0.47; 0.87]
Total (fixed effect)		100	0.70	[0.61; 0.78]
Total (random effects)			0.70	[0.61; 0.78]
Heterogeneity: $\chi^2_4 = 1.99$ ($P = .74$), $I^2 = 0\%$				
Publication type = article				
Goss 2018	27	50	0.54	[0.39; 0.68]
Reungwetwattana 2018	20	22	0.91	[0.71; 0.99]
Wu 2018	21	30	0.70	[0.51; 0.85]
Xie 2019	10	31	0.32	[0.17; 0.51]
Xing, P 2019	8	15	0.53	[0.27; 0.79]
Total (fixed effect)		148	0.65	[0.58; 0.72]
Total (random effects)			0.61	[0.39; 0.82]
Heterogeneity: $\chi^2_4 = 36.69$ ($P < .001$), $I^2 = 89\%$				
Total (fixed effect)		248	0.67	[0.61; 0.72]
Total (random effects)			0.64	[0.53; 0.76]
Heterogeneity: $\chi^2_9 = 39.29$ ($P < .001$), $I^2 = 77\%$				
Residual heterogeneity: $\chi^2_8 = 38.68$ ($P < .001$), $I^2 = 79\%$				

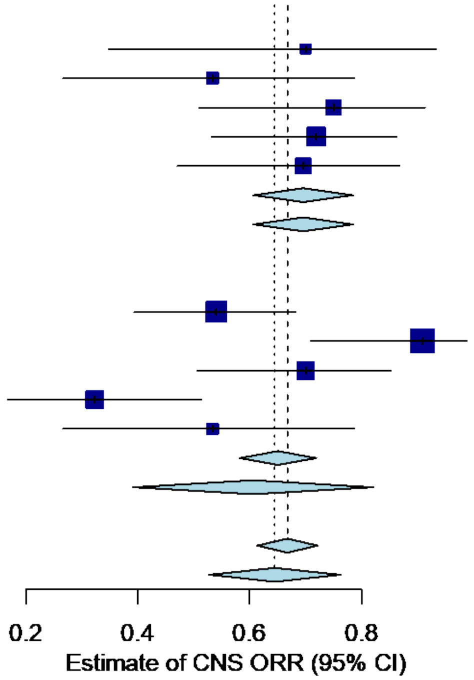

© 2020 Erickson AW et al. *JAMA Network Open*.

eFigure 10. Forest plot of reported CNS DCR, stratified by retrospective versus prospective.

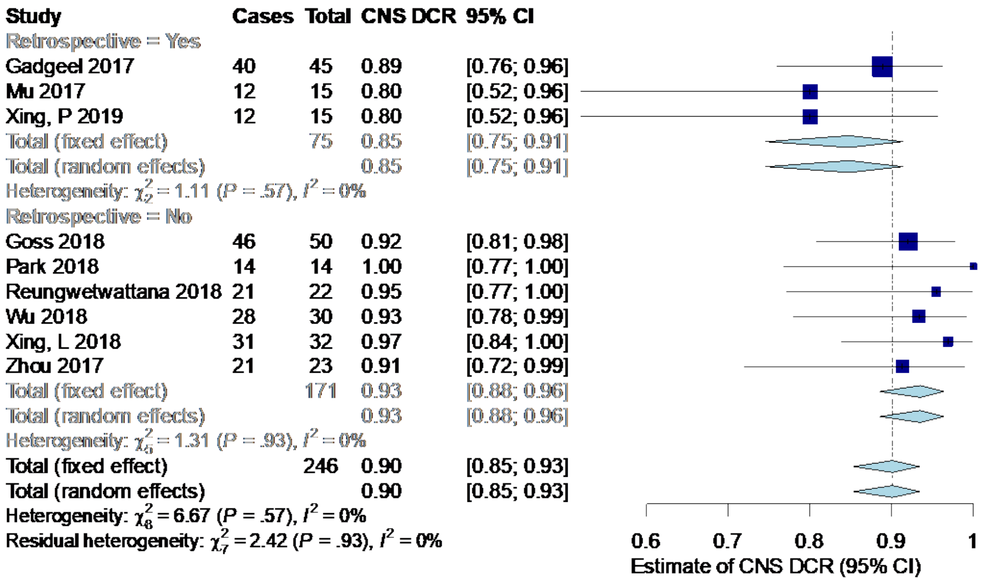

© 2020 Erickson AW et al. *JAMA Network Open*.

eFigure 11. Forest plot of reported CNS DCR, stratified by line of therapy.

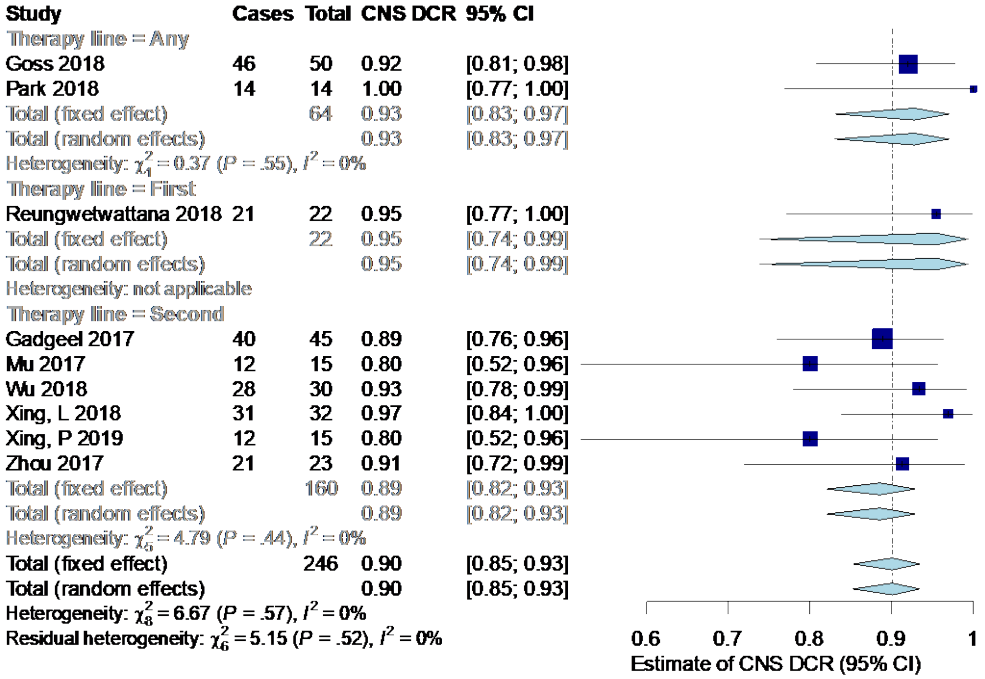

© 2020 Erickson AW et al. *JAMA Network Open*.

eFigure 12. Forest plot of reported CNS DCR, stratified by pharmaceutical industry funding.

Study	Cases	Total	CNS DCR	95% CI
Pharmaceutical funding = No				
Park 2018	14	14	1.00	[0.77; 1.00]
Xing, P 2019	12	15	0.80	[0.52; 0.96]
Total (fixed effect)		29	0.85	[0.64; 0.95]
Total (random effects)			0.88	[0.55; 0.98]
Heterogeneity: $\chi_1^2 = 1.58$ ($P = .21$), $I^2 = 37\%$				
Pharmaceutical funding = NR				
Gadgeel 2017	40	45	0.89	[0.76; 0.96]
Mu 2017	12	15	0.80	[0.52; 0.96]
Total (fixed effect)		60	0.86	[0.75; 0.93]
Total (random effects)			0.86	[0.75; 0.93]
Heterogeneity: $\chi_1^2 = 0.75$ ($P = .39$), $I^2 = 0\%$				
Pharmaceutical funding = Yes				
Goss 2018	46	50	0.92	[0.81; 0.98]
Reungwetwattana 2018	21	22	0.95	[0.77; 1.00]
Wu 2018	28	30	0.93	[0.78; 0.99]
Xing, L 2018	31	32	0.97	[0.84; 1.00]
Zhou 2017	21	23	0.91	[0.72; 0.99]
Total (fixed effect)		157	0.93	[0.88; 0.96]
Total (random effects)			0.93	[0.88; 0.96]
Heterogeneity: $\chi_4^2 = 1.06$ ($P = .90$), $I^2 = 0\%$				
Total (fixed effect)		246	0.90	[0.85; 0.93]
Total (random effects)			0.90	[0.85; 0.93]
Heterogeneity: $\chi_8^2 = 6.67$ ($P = .57$), $I^2 = 0\%$				
Residual heterogeneity: $\chi_6^2 = 3.39$ ($P = .76$), $I^2 = 0\%$				

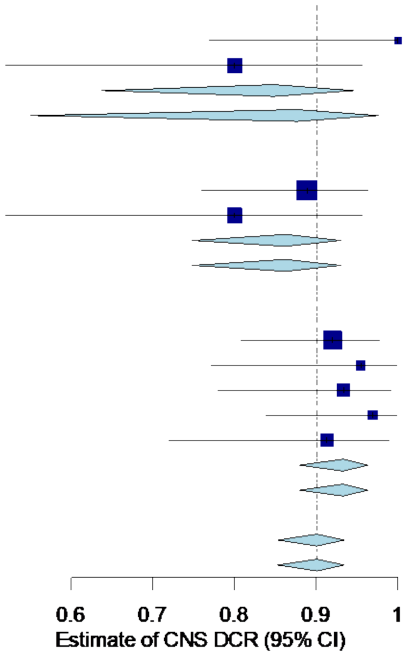

© 2020 Erickson AW et al. *JAMA Network Open*.

eFigure 13. Forest plot of reported CNS DCR, stratified by randomized controlled trials versus other study types.

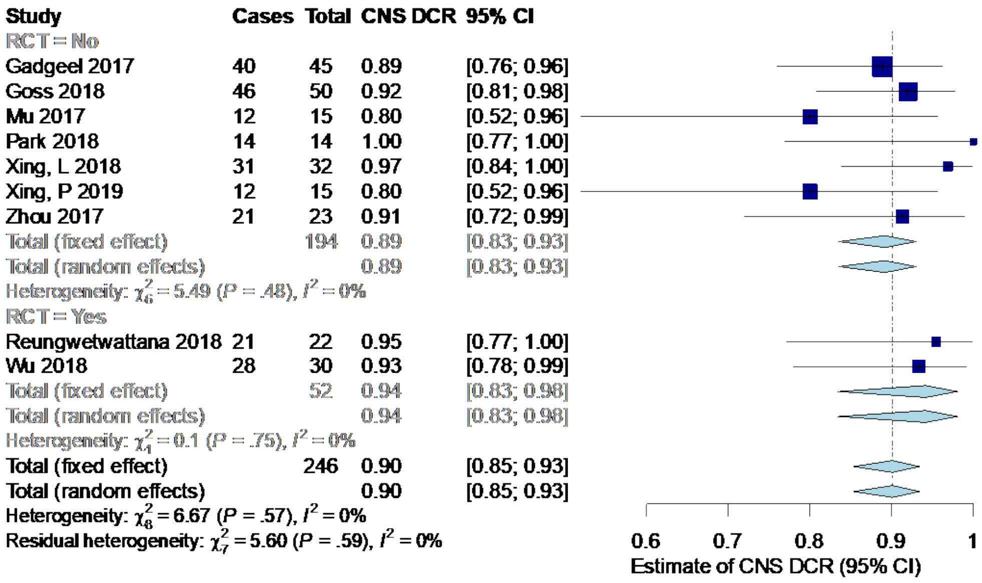

eFigure 14. Forest plot of reported CNS DCR, stratified by abstract versus article.

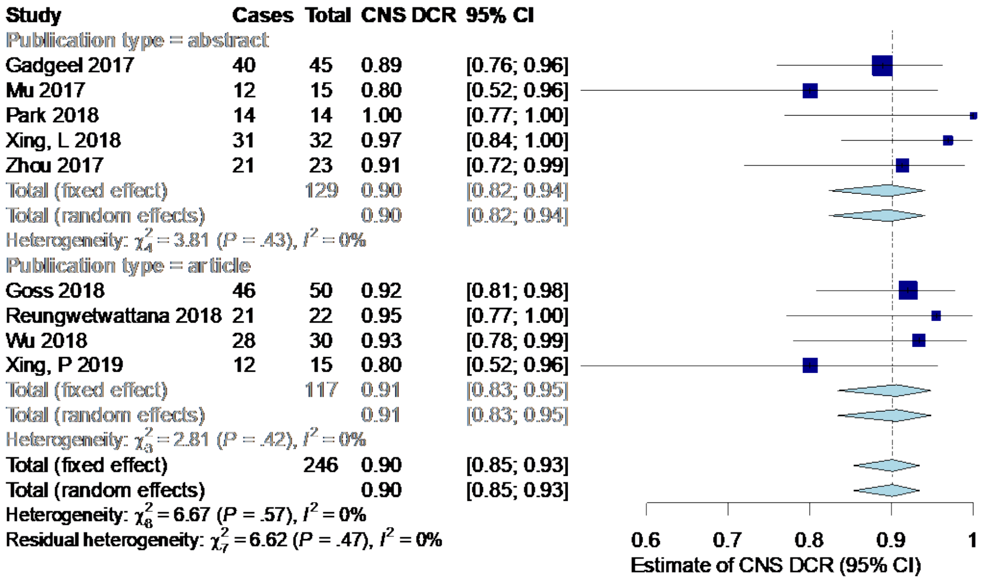

© 2020 Erickson AW et al. *JAMA Network Open*.

eFigure 15. Forest plot of risk ratio for CNS ORR among included randomized controlled trials.

Source	Comparator	RR (95% CI)
Reungwetwattana, 2018	Gefitinib or erlotinib	1.33 [0.95; 1.85]
Wu, 2018	Platinum-pemetrexed	2.24 [1.04; 4.81]
Total		1.44 [1.06; 1.96]

Heterogeneity: $\chi^2_1 = 1.51$ (P = .22), I^2 = 34%

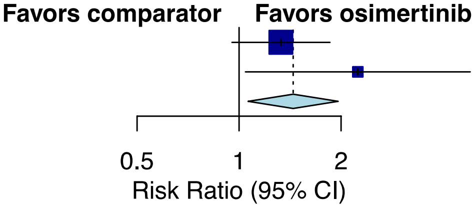

© 2020 Erickson AW et al. *JAMA Network Open*.

eFigure 16. Forest plot of risk ratio for CNS DCR among included randomized controlled trials.

Source	Comparator	RR (95% CI)
Reungwetwattana, 2018	Gefitinib or erlotinib	1.07 [0.89; 1.28]
Wu, 2018	Platinum-pemetrexed	1.49 [1.01; 2.21]
Total		1.13 [0.96; 1.33]

Heterogeneity: $\chi^2_1 = 2.34$ (P = .13), I^2 = 57%

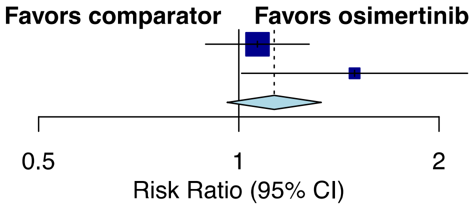

eFigure 17. Cochrane risk of bias tool assessment of phase III studies. All four studies identified as phase III were assessed using the Cochrane RoB 2 tool. Two phase III studies did not include comparator groups and were not eligible for assessment of bias in randomization or allocation concealment.

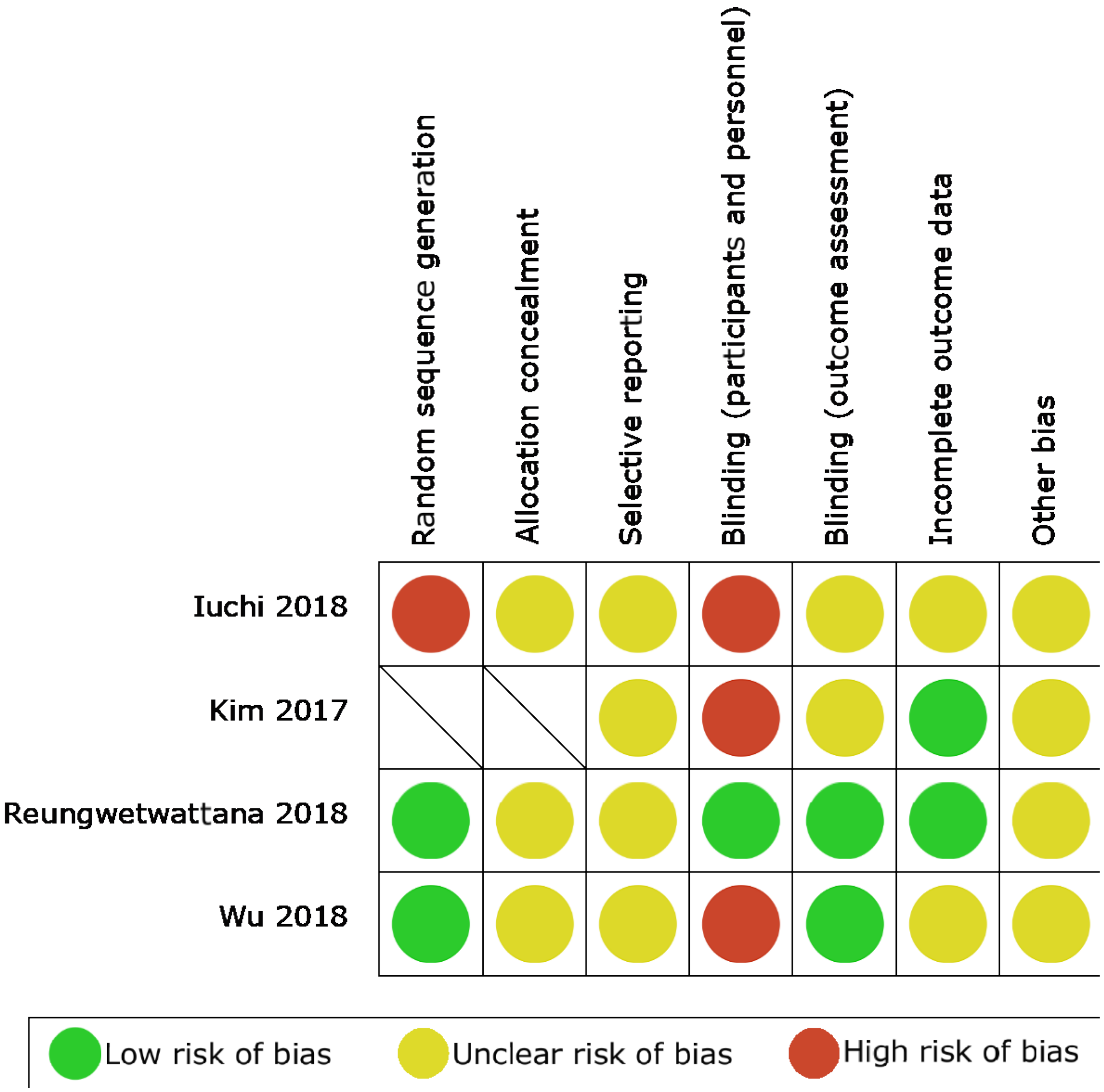

© 2020 Erickson AW et al. *JAMA Network Open*.

eFigure 18. Modified Newcastle-Ottawa scale assessment of phase II and retrospective studies. The Newcastle-Ottawa scale for cohort studies was modified with the subtraction of three questions—two relating to selection and comparison of non-exposed patients and one assessing presence of outcome at study start—and the addition of one question addressing presence of pharmaceutical industry funding.

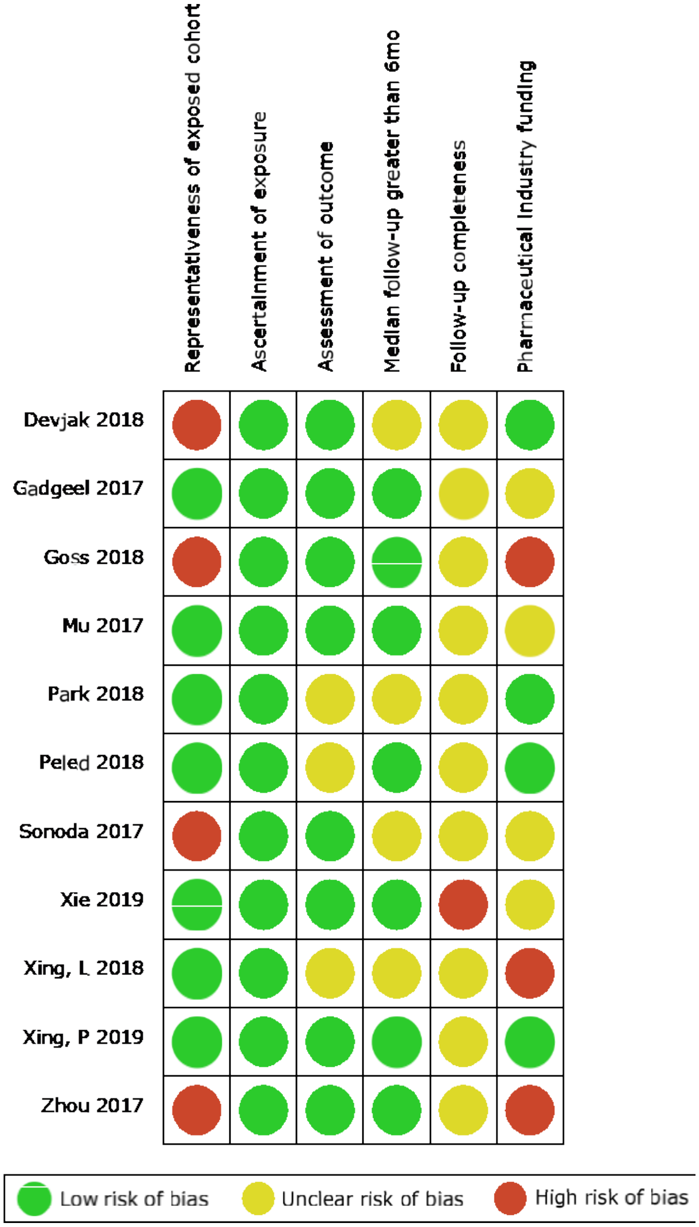

eReferences.

1. Higgins JP, Thompson SG, Deeks JJ, Altman DG. Measuring inconsistency in meta-analyses. *BMJ.* 2003;327(7414):557-560.
2. Wang N. Conducting Meta-Analyses of Proportions in R. In: Texas A&M University; 2018.
3. Wu YL, Ahn MJ, Garassino MC, et al. CNS efficacy of osimertinib in patients with T790M-positive advanced non-small-cell lung cancer: Data from a randomized Phase III trial (Aura3). *Journal of Clinical Oncology.* 2018;36(26):2702--2709.
4. Reungwetwattana, T, Nakagawa K, et al. CNS response to osimertinib versus standard epidermal growth factor receptor tyrosine kinase inhibitors in patients with untreated EGFR-mutated advanced non-small-cell lung cancer. *Journal of Clinical Oncology.* 2018;36(33):3290--3297.
5. *R: a language and environment for statistical computing.* [computer program]. Vienna, Austria: R Foundation for Statistical Computing; 2019.
6. Viechtbauer W. Conducting meta-analyses in R with the metafor package. *Journal of Statistical Software.* 2010;36(3):1–48.
7. Schwarzer G. meta: an R package for meta-analysis. *R News.* 2007;7(3):40–45.
8. Sterne JAC, Savović J, Page MJ, et al. RoB 2: a revised tool for assessing risk of bias in randomised trials. *BMJ.* 2019;366:l4898.
9. Wells G, Shea B, O'Connell D, et al. The Newcastle-Ottawa scale (NOS) for assessing the quality of nonrandomised studies in meta-analyses. 2009. Available from: http://www.ohri.ca/programs/clinical_epidemiology/oxford.asp. Accessed Oct 9, 2019. In.
10. Devjak R, Hitij NT, Mohorcic K, Rajer M. CNS response to osimertinib in patients with EGFR mutated lung adenocarcinoma: Real world data. *Journal of Thoracic Oncology.* 2018;13(4 Supplement 1):S92--S93.
11. Gadgeel S, Chen W, Piotrowska Z, et al. Clinical activity of osimertinib in EGFR mutation positive non-small cell lung cancer (NSCLC) patients (Pts) previously treated with rociletinib. *Journal of Thoracic Oncology.* 2017;12(1 Supplement 1):S1263.
12. Goss G, Tsai CM, Shepherd FA, et al. CNS response to osimertinib in patients with T790M-positive advanced NSCLC: pooled data from two phase II trials. *Ann Oncol.* 2018;29(3):687-693.
13. Iuchi T, Sakaida T, Hasegawa Y, et al. Brain metastases from EGFR-mutated NSCLC which had acquired resistance to EGFR-TKI. \~ lessfrequent T790m and preserved response to other TKIS \~. *Neuro-Oncology.* 2018;20(Supplement 6).
14. Kim JH, Kim HR, Hong MH, et al. Efficacy of osimertinib for brain metastasis in advanced NSCLC: Data from single center in ASTRIS Trial. *Journal of Thoracic Oncology.* 2017;12(11 Supplement 2):S2211.
15. Mu Y, Xing P, Hao X, Wang Y. A Retrospective Study: Central Nervous System Response to Osimertinib in Patients with Advanced NSCLC. *Journal of Thoracic Oncology.* 2018;13(10 Supplement):S982--S983.

© 2020 Erickson AW et al. *JAMA Network Open*.

16. Park C, Cho H, Choi YD, Oh I. Osimertinib in the First-Line Treatment of Non-Small Cell Lung Cancer Harboring Activating EGFR Mutation from Circulating Tumor DNA. *Journal of Thoracic Oncology.* 2018;13(10 Supplement):S492.
17. Peled N, Rotem O, Rozenblum A, et al. Osimertinib for EGFR-Positive Advanced NSCLC with Brain Metastases: Preliminary Analysis of an Open-Label, Two-Arm, Phase 2 Study. *Journal of Thoracic Oncology.* 2018;13(10 Supplement):S665--S666.
18. Sonoda T, Yanagitani N, Saiki M, et al. The efficacy and toxicity of osimertinib in T790M-positive NSCLC with acquired resistance to EGFR-TKI in clinical practice. *Journal of Clinical Oncology.* 2017;35(15 Supplement 1).
19. Xie L, Nagpal S, Wakelee HA, et al. Osimertinib for EGFR-Mutant Lung Cancer with Brain Metastases: Results from a Single-Center Retrospective Study. *Oncologist.* 2019;24(6):836--843.
20. Xing L, Pan Y, Shi Y, et al. Efficacy and Safety of Osimertinib in EGFR T790M-Positive Advanced NSCLC Patients with Brain Metastases (APOLLO Study). *Journal of Thoracic Oncology.* 2018;13(10 Supplement):S592 [abstract].
21. Xing P, Mu Y, Hao X, Wang Y, Li J. Data from real world to evaluate the efficacy of osimertinib in non-small cell lung cancer patients with central nervous system metastasis. *Clinical and Translational Oncology.* 2019.
22. Zhou C, Cheng Y, Lu Y, et al. CNS response to osimertinib in Asian-Pacific patients (pts) with T790M-positive advanced NSCLC: Data from an open-label Phase II trial (AURA17). *Annals of Oncology.* 2017;28(Supplement 5):v484.

© 2020 Erickson AW et al. *JAMA Network Open*.
